# “Nothing Special, Everything Is *Maamuli*”: Socio-Cultural and Family Practices Influencing the Perinatal Period in Urban India

**DOI:** 10.1371/journal.pone.0111900

**Published:** 2014-11-04

**Authors:** Shanti Raman, Krishnamachari Srinivasan, Anura Kurpad, Husna Razee, Jan Ritchie

**Affiliations:** 1 Department of Community Paediatrics, South Western Sydney Local Health District, Liverpool Hospital, Liverpool, New South Wales, Australia; 2 Department of Psychiatry, St John’s Medical College, Bangalore, India; 3 Department of Physiology, St John’s Medical College, Bangalore, India; 4 School of Public Health & Community Medicine, University of New South Wales, Sydney, New South Wales, Australia; Queensland University of Technology, Australia

## Abstract

**Background:**

Globally, India contributes the largest share in sheer numbers to the burden of maternal and infant under-nutrition, morbidity and mortality. A major gap in our knowledge is how socio-cultural practices and beliefs influence the perinatal period and thus perinatal outcomes, particularly in the rapidly growing urban setting.

**Methods and Findings:**

Using data from a qualitative study in urban south India, including in-depth interviews with 36 women who had recently been through childbirth as well as observations of family life and clinic encounters, we explored the territory of familial, cultural and traditional practices and beliefs influencing women and their families through pregnancy, childbirth and infancy. We found that while there were some similarities in cultural practices to those described before in studies from low resource village settings, there are changing practices and ideas. Fertility concerns dominate women’s experience of married life; notions of gender preference and ideal family size are changing rapidly in response to the urban context; however inter-generational family pressures are still considerable. While a rich repertoire of cultural practices persists throughout the perinatal continuum, their existence is normalised and even underplayed. In terms of diet and nutrition, traditional messages including notions of ‘hot’ and ‘cold’ foods, are stronger than health messages; however breastfeeding is the cultural norm and the practice of delayed breastfeeding appears to be disappearing in this urban setting. Marriage, pregnancy and childbirth are so much part of the norm for women, that there is little expectation of individual choice in any of these major life events.

**Conclusions:**

A greater understanding is needed of the dynamic factors shaping the perinatal period in urban India, including an acknowledgment of the health promoting as well as potentially harmful cultural practices and the critical role of the family. This will help plan culturally appropriate integrated perinatal health care.

## Introduction

Health conditions affecting the perinatal period still account for a major contribution to disease burden in sub-Saharan Africa and South Asia despite the significant global shift in disease burden towards non-communicable diseases [1], [2], making the perinatal period i.e. pregnancy, childbirth and infancy, a key period for health intervention. Of this global burden of maternal and neonatal deaths, India contributes the largest share [3]–[6]. India also contributes the largest share in terms of sheer numbers, of maternal and infant under-nutrition, starting with low birth weight infants [7]. Public health approaches to maternal and newborn health over the last decade have emphasized the need for access to services, chiefly skilled attendance and emergency obstetric care, but have paid comparatively less attention to improving family and community practices [8]. A major gap in our knowledge on how to improve perinatal outcomes, is how family and community practices influence maternal/child health care-seeking behaviours [9]. Given India’s significant contribution to global maternal and infant mortality, a focus on this country is integral to any successful global effort [10].

Over the past two decades there has been considerable interest in understanding the socio-cultural milieu of pregnancy and childbirth in low-and middle-income countries so that interventions can be culturally appropriate and have a greater chance of success. The majority of the research has centred on ‘traditional’ or cultural care practices around birth and delivery and the newborn period; and much of it is based on rural populations [11]–[13]. There has been understandable emphasis in the research on potentially harmful traditional practices, particularly in settings such as in rural South Asia and sub-Saharan Africa, where home deliveries are common. We know from recent research in rural India that unhygienic cord cutting, delayed breastfeeding and early bathing [14], and a combination of traditional and modern practices rooted in the concept of inducing heat to facilitate labour, continue to take place [12]. There has also been robust research aimed at describing early infant nutrition, particularly cultural approaches to breastfeeding initiation and duration [15]–[18].

Less understood and described and, we would argue no less important, are the socio-cultural milieu and practices across the whole of the perinatal continuum and in the urban setting. Darmstadt et al. [19] reviewed the literature of antenatal, intra-partum, and postpartum care practices for mothers and newborns in Bangladeshi communities and found a dearth of information. Studies of newborn care practices in the slums of Dhaka [20], and Karachi [21], have found that these were similar to those in rural areas of the South Asia region, including lack of exclusive breastfeeding, bathing the baby soon after birth and applying substances to the umbilical cord. During the 1990s, the MotherCare Project conducted qualitative research to determine the major barriers and facilitators of iron supplementation programs for pregnant women in eight developing countries; beliefs against consuming medications during pregnancy, and fears that taking too much iron may cause too much blood or a big baby, making delivery more difficult were common [22]. In summary, research has been more focused on the potentially dangerous cultural practices during pregnancy, childbirth and newborn period, and less interested in the health promoting aspects of cultural practices.

In 2003 a prospective birth cohort study was commenced at St John’s Medical College (SJMC) Hospital, Bangalore, India, to explore the association of maternal health and nutrition with pregnancy and child health outcomes. Several salient reports and results have already emerged out of this cohort study [23], [24]; in keeping with international research findings, the single most important factor in determining birth weight and hence infant outcomes in this cohort was maternal education level [25]. We situated our qualitative study within this cohort to elicit psychosocial and cultural factors that influence the perinatal period, for mother and infant dyads in urban India. In particular we wanted to explore how ‘cultural’ practices and beliefs influenced women’s pregnancy and childbirth experiences, their pregnancy and family planning choices, their self-care including nutrition through the perinatal continuum.

## Empirical Setting, Methods and Data

This paper draws on ethnographic work carried out in greater metropolitan Bangalore, (now Bengaluru), a contemporary urban landscape in India which includes large areas that were until recently considered villages (rural), but have become incorporated into the city. Our previous paper focused on sources of support available to mother-infant dyads [26], we have also reported on the challenges of accessing healthcare in the perinatal period [27]. In this paper we report specifically on the role cultural ideas, beliefs and practices play in influencing the perinatal period in the urban setting.

SJMC Hospital is a 1200-bed tertiary healthcare service in Bangalore, which draws patients of diverse socioeconomic status, from urban slums to high-income residential areas. For the in-depth interviews, we identified women from the cohort who had been through pregnancy and childbirth within the last two years. We used maximum variation sampling [28], to ensure a mix of social and cultural groups (i.e. language and religion) from within three education levels from the cohort. These included women with low education levels (primary school-Group 1), women with medium education levels (completed high school-Group 2) and women with high education levels (tertiary education – Group 3). Prior to commencing the interviews, an interview guide was formulated based on a literature review including topics such as home environment, sources of support, pregnancy and childbirth expectations including gender preference, choice and control over reproduction, cultural practices, dietary practices and self-care. Participants were initially contacted by telephone to locate them, as most addresses were incomplete or wrong; the mobile phone proving the most useful method of reaching even the most unreachable. None of the women refused to participate; some were unable to be located due to change in mobile phone number. Participants were interviewed by female researchers (the first author and research assistant) in the location of their choice; most often it was their home, sometimes it was their mother’s house, or their in-laws’ house, occasionally it was their workplace. While the interview subjects were recent mothers, due to the ethnographic approach taken and the reality of conducting qualitative research in India, often the extended family or even friendship network participated. Interviews were continued till a saturation of themes was reached. Each in-depth interview lasted between 1.5 hours to two hours.

Ethnographic observations were carried out during fieldwork by the first author between August 2008 to January 2009 and in December 2010 in Bangalore. Observations were carried out during formal and informal encounters in family homes or workplaces and while interacting with the extended family and friendship network of the participants or during maternal and child antenatal and postnatal healthcare visits in SJMC and a government health centre. The ethnographic approach was influenced strongly by active listening [29], and used a range of methodologies as described by Fitzgerald including observation, participation, formal and informal interviewing and critical self- reflection [30].

Audio taped interviews were transcribed (from the language of interview to English) as soon as possible following the interview either by the first author or the research assistant (RA) and verified by each other. Observation notes were recorded as field notes in a journal and reflective observations entered in Microsoft Word. The transcripts were open-coded manually and re-categorised by the first author and the RA [28]. One other member of the research team in Bangalore independently read all the transcripts and cross-coded the interviews to ensure data integrity Data were analysed using thematic analysis and constant comparative techniques [31]. Overall analysis was iterative, being guided by the principles of grounded theory and phenomenology and incorporating critical reflexivity [32].

### Ethics statement

Ethics clearance was obtained from the Institutional Ethical Review Board of the St John’s Medical College and Hospital prior to commencing the fieldwork. Informed consent was read and translated to the language of choice by the first author or RA; all participants provided written consent through a signature or initials in English or in Kannada.

## Results

In all, in-depth interviews were carried out with 36 women, 13 in group one, 12 in group two, 11 in group three. Table 1 summarises characteristics of those interviewed. The results of the analysis and interpretation of the qualitative data are presented as key themes identified with respect to the research question: how do cultural practices and beliefs impact on the perinatal continuum for maternal-infant dyads in Bangalore? The following themes emerged:

**Table 1:** Characteristics of participants, perinatal socio-cultural study, Bangalore.

	Group 1 Low education	Group 2 Medium education	Group 3 Tertiary education
Number	13	12	11
Languages spoken	6	5	5
Maternal age (mean)	23 years	24 years	26 years
Baby birth weight (mean)	2.6 kg	2.7 kg	3.1 kg
Infant outcomes	1 death, 1 disability, 2 stunted	1 stunted, 1 mild disability	Good overall

**1. Pregnancy related expectations and experiences**
  - **Fertility concerns**
  - **Gender preference**
  - **Desire for small family**
  - **Reproductive choice**

**2. Traditional cultural practices**
**3. Dietary practices through perinatal period**

### Pregnancy related expectations and experiences

#### *I was very “tense” because I did not get pregnant: Fertility concerns*

An overwhelming concern for many women, who were asked about their pregnancy, was around demonstrating their fertility early, following marriage. This cut across education levels, religious and cultural affiliations. Many women when asked about details about pregnancy would recount their problems with ‘falling pregnant’ first. As 27 year old Priya (names have been changed throughout to preserve anonymity) who worked as a teacher recounted, “I was very tense [English word used] because I did not get pregnant.” Priya and her husband had been trying to get pregnant for two years ever since she got married, “after the [fertility] treatment and many tests” the couple were successful in their quest. Thirty year old Sumathi, related with some distress the hazardous journey that fertility and reproductive services had been for her. Sumathi and her husband had lost their first son in a tragic drowning accident in their village. After some difficulty, the couple had a girl. Following this, the extended family said “get another baby”. Although her husband was happy with one child, family pressure prevailed. “I have had lots of investigations and ultrasounds, *tumbane* (too much) problem.” The couple had spent a lot of money and waited seven years for another successful pregnancy. After five miscarriages in as many years, Sumathi finally had a successful pregnancy; she related that her doctor had said “we have tried our best; the rest is up to God.”

Very often the question about whether the pregnancy was planned would be interpreted as a query regarding family planning or contraception use. Invariably women would offer up their accounts of their fertility concerns. As 20 year old Shashi with limited high school education said,

I thought of having three years [*family*] planning. But my in-laws started acting differently towards me as I was not pregnant. Due to their forcing I got pregnant so soon (i.e. within three months).

#### *Only girls look after their families: Gender preference*

Contrary to our expectations most women asked about gender preference in their pregnancy expressed a strong desire for a girl, especially if it was the first pregnancy. None of the women we spoke to admitted to any knowledge during the pregnancy of the expected gender of the baby. As typified by Ganga, a working woman with low education levels, who at 31 years was one of the oldest women in the cohort, said:

We both wanted girl child only. Only girls look after their families, boys are a waste. Girls understand family issues better, that is why we like girls. I want only one girl, and I want to give her a good education.

Women, as well as their husbands if they were around, reflected a preference for girls. Some women acknowledged that their families, more importantly their mothers-in-law, expected a boy. As 20 year old Shashi said, “for my in-laws’ sake I was expecting for boy…. but I preferred girl only. I am very happy with a girl now.”

While all women denied any ultrasound-assisted gender identification prenatally, many women spoke of having ‘abortions’. Sometimes this term related to spontaneous miscarriages, other times it was specifically used to denote medical terminations of pregnancy. Twenty year old Shobha who had eloped to have a ‘love marriage’ and had suffered from financial stresses in the past said, “Actually I had two abortions between first and second child. This time we were happy about pregnancy, both husband and I wanted a girl.”

Women who expressed a preference for a boy were in a minority and usually had strong family pressure bearing down on them. The two women who had a strong preference for a boy were both Muslim, and already had two girls each. As Shabnam (29 years old, educated, teacher) said:

I really, really wanted a boy after two girls. I had a lot of health problems associated with pregnancy, in the past. I was told **not** to become pregnant (emphatic hand gestures), by doctors and all. But so much I was praying for a boy.

Shabnam also admitted that even her husband had suggested she go for an abortion, given her past health problems. However her mother supported her throughout this pregnancy saying, “I have prayed for you, this time it will be different, you *will* have a boy.” Only one other young mother Kala, a 22 year old with little education expressed a desire for a boy with this pregnancy, she also already had two daughters. Her mother, who often spoke in her stead, said, “we thought if it is a boy, she can go for family planning [*referring to tubal ligation*], but now [*meaning the next pregnancy*] she will try for a boy.”

#### *We are not planning for another one: Desire for small, healthy family*

Another strong sentiment expressed, often unsolicited was for a small, healthy family. Many families were living in cramped one room dwellings. The emphasis seemed to be on making sure that the family unit was able to provide adequately for and bring up optimally, a finite number of children. Only three women we spoke to were even contemplating having more than two children. A surprising minority expressed a preference for having only one child; this view was expressed by Hindus and Christians across class divides, but never by Muslim women. “We just wanted a healthy baby”, was oft repeated, the emphasis being on ‘healthy’. As 26 year old Anita (Christian, poorly educated), who already had one boy said, “We are not planning for another one; because in our family all are telling us, next will be a boy only.” Ganga who had expressed strong views about the benefits of having girls, was preparing to have her “family planning operation.” Educated and well off Sumithra at 25 years of age and with one daughter said, “No, one is enough, I feel tired to look after this child only.” Women often said, “I feel one child is enough, but my husband wants another.”

#### *We did some planning for two years: Reproductive choice*

We did not specifically probe about whether women had control over their fertility or reproductive choice. Various questions and observations were analysed to gather information about women’s choice in fertility and reproduction. Choice and control over reproduction was linked to choice of marriage partner and ability to make choices regarding fertility. For example, Shobha who had primary school education only and came from a poor rural family had defied tradition to marry a man from her village across caste lines. The couple were isolated from both their families of origin. Despite her young age, at 21 years, Shobha had decided, “I am not having any more pregnancies; I will get operated on soon”. Twenty-four year old Pooja, who had also had a love marriage, and insisted that they had planned the pregnancy, when asked when she actually got pregnant replied happily, “After one month of my marriage I got pregnant.” Twenty-eight year old Grace, Catholic with tertiary education also admitted to a ‘love marriage’. Grace worked as a marriage counsellor; she followed birth spacing and Billings method for contraception, “as I give advice on such things only in my counselling.”

Love marriages were an exception; most of the women we interviewed had arranged marriages. At least half of the women particularly from the less educated groups originally hailed from villages in South India and endogamy was widely practised (cross-cousin and maternal uncle marriages common). Mumtaz, who was poorly educated, yet was extremely confident in the social sphere, said she and her husband did “some (family) planning for two years” prior to the first successful pregnancy at 25 years. About half the women in the less educated groups said they had some choice and control over their reproduction and fertility, women usually referred to this as ‘planning’ (English term used). Mostly women said that they did some planning for two years, rarely longer than that. Among highly educated women, more of them said they had some measure of control over their planning. However those that had the least say in their marriage choice likewise had little choice or control over their fertility. Twenty-two year old Sameena (Muslim) got married “within one week after finishing my exams” and admitted, “We didn’t have any planning, after two months of my marriage I got pregnant.”

#### *Nothing special. Everything is “maamuli” only: Traditional (cultural) practices*

Most women in their first pregnancy described having a religious or cultural ritual in the third trimester to celebrate the mother and the pregnancy; this was celebrated by Hindus, Muslims and Christians in some manner. However the question of whether women did anything special during their pregnancy was often responded to with, “nothing special, everything is *maamuli* (ordinary) only”; suggesting that pregnancy and birthing rituals and other practices were commonplace and were nothing out of the ordinary. The South Indian term for the special ritual in pregnancy is *Seemantha* or *Vallécāpu*, and is celebrated during a woman’s first pregnancy, in preparation for her first delivery; it is a rite of passage into motherhood. In many cases the ritual is performed in the woman’s in-laws’ house, but the bulk of expenses are expected to be borne by her own natal kin. It is commonly performed as a ritual celebrating fertility and the mother, various emic symbols are used as presents and offerings to the mother-to-be including bangles, coconuts and special saris. It is customary for the pregnant woman to return to her own mother’s house following the ritual and remain with her mother for the customary six weeks or three months post-delivery. Despite the expectations of following this script, in reality the *Seemantha* or equivalent ceremony was celebrated in a range of ways in Bangalore. For example, Savitha who had an early pregnancy miscarriage previously was being specially cared for at her own parents’ (natal) home. This lasted from mid pregnancy through to almost 9 months post baby’s birth. “Yes, here they arranged *seemantha* for me, my husband’s family bought everything here (to her natal home) and they did the ceremony.” Twenty-four year old Pooja and her husband, came from North-eastern India and had no family in Bangalore. Pooja admitted to missing her mother and her natal family very much through her pregnancy. However, neighbours in this crowded and dilapidated housing complex had taken on family responsibility for Pooja and her husband, despite being from different cultural and language backgrounds. The husband gratefully described, “Our neighbours arranged function in the next building. I got a priest to do *puja* (religious ritual); rest all they did.” Mumtaz, 25 year old Muslim woman with primary school education, said “We had a seven month function (using English word) here (at husband’s place).”

Many women talked of missing the ceremony if it wasn’t performed. Shobha who was isolated from her natal family “because I did love marriage”, said regretfully, “We did *Seemantha* for the first pregnancy, but nothing special this time. I did not go to mother’s place even; I feel my mother does not have much love for me.” Sometimes the ritual was avoided because of medical complications, “We did nothing special cultural, mainly followed medical advice, as this was a very precious pregnancy.” Sometimes it was due to family tension or financial tensions. As with 20 year old Shashi who related that her parents requested her in-laws, “shall we arrange a function?” but was told that they (in-laws) “didn’t have time for all those things.” This was a family with major in-law tensions; exacerbated by the fact that the couple’s marriage was endogamous (related).

Specific cultural practices for the infant were also commonly performed, but unless specifically asked about, not mentioned. A range of naming ceremonies or specific religious ceremonies was carried out in early infancy, depending on cultural group. As 23 year old Mary said, “For the baby, he was baptized and we did ‘holy communion’; we also recently went to Velankanni temple and offered baby’s hair as thanksgiving.” Most infants wore charms, talismans (*rakshé* or *tabiz*), or religious icons around their waists or necks, many also had beauty spots (black mark) on the face to ward off the evil eye.

#### *Cool foods and ‘heaty’ foods: Dietary practices in pregnancy and postnatally*

Women and their families had strongly held cultural views about food and nutrition. While all the women were part of the larger cohort study and were being asked questions about their diet and provided dietary advice in pregnancy; they most often spoke about cool foods and heaty foods and the effect these had on their pregnancy. Anita a 25 year old woman with little education said, that the “doctor told me eat whatever you want, but avoid ‘heaty’ food.” She also said that many of her family and friends had suggested that she “avoid cool food” and “have heat food” instead. Others were equally adamant that they must avoid ‘heaty’ foods. Thirty-two year old Ganga who had a high school education level and strongly held views said, “Doctor suggested me to take lot of spinach, fruits, fish; but I was avoiding *musumbi* [sweet lime] as it causes cold effect on me. I was avoiding papaya, as it is ‘heaty.’” Most women had some idea about whether their body type was ‘heaty’ or ‘cool’. Radhika, who was well educated and even worked for a health firm in the past, said “I used to avoid papaya, because mine is a ‘heat’ body. Whenever I ate papaya, the next day only I used to get urine problems.” None of the women admitted to restricting their diet to have a small baby or facilitate easy delivery. Some women spoke about special foods that were made for them usually by their mothers, during pregnancy and also for the immediate post-natal period; the nutritional content was never mentioned. *Nāti aushadi* or folk medicine was also mentioned, as part of the diet particularly to combat nausea in pregnancy. All the women as part of the cohort were given iron and calcium supplements throughout pregnancy and the early postnatal period, most women said they took these tablets. However when they described their diets, it was mostly about the fit between the foods and their body type rather than the nutritional content. Twenty-five year old Mumtaz (Muslim, poorly educated), was the only one who specifically mentioned taking rest in pregnancy and also being supported by the family to take rest. Her desire was to have a ‘fair child’, “everyone told me to eat two apples daily and half litre of milk, I did that daily; that’s why I have such a beautiful, fair baby (laughing)!”.

Breastfeeding was universally practised, all had initiated breastfeeding early. When asked specifically about breastfeeding initiation, the usual response was “soon after delivery”. Apart from one woman, who did say she needed some help and support from her mother with establishing breastfeeding, there were none who reported problems. Baby massages with application of oil was also common. Another universally common practice was ensuring clean water for the infant, usually “I boil water for baby”. Even where families had difficulties accessing adequate water supply, they would ensure that any water used to feed or cook for the infant was boiled. Complementary feeds for the infant were usually traditional foods, slightly modified according to the age of the child, but not by cultural group. “Morning we are giving rusk, *dosa* or *idli* (rice and lentil based local food), also we are giving him almonds, *ragi* (local millet) malt.” This description of complementary feeding was repeated many times and by women from all cultural groups.

## Discussion

This qualitative study explores the socio-cultural factors that shape the perinatal period in urban India today. Public health researchers and policymakers are increasingly acknowledging the need to understand pregnancy and childbirth beyond morbidity and mortality statistics [10], [33]. More than two decades ago Cleland and van Ginneken suggested that the intervening role of cultural beliefs and domestic practices may be very important in the explanation of the maternal education-perinatal mortality relationship [34]. We found that while there were many similarities in cultural practices and beliefs in South Asian anthropological studies [35]–[39], there are changing practices and ideas that deserve documenting and recognising. We found in our study that fertility concerns dominate women’s experience of married life; that notions of ideal family size and gender preference are changing rapidly in response to the urban context; that while a rich repertoire of cultural practices persist throughout the perinatal continuum their existence is normalised and even underplayed; and that in terms of diet and nutrition traditional messages are stronger than health messages. Marriage, pregnancy and childbirth are so much part of the norm for women that there is little expectation of individual choice in any of these major life events. The family unit, including extended family, exerts a critical influence throughout the perinatal period, as noted previously [26].

There has been acknowledgement that infertility is an important public health issue [40], [41]; the International Conference on Population and Development Programme of Action stating that reproductive health services should include the prevention and appropriate treatment of infertility [42]. Yet this common problem has received little attention from health policy makers in India. While the World Health Organization recommends the epidemiological definition of infertility, which is the inability to conceive within two years of exposure to pregnancy [43], we found that women in our study felt pressured to demonstrate their fertility soon after they got married, and if that did not occur concerns about infertility dominated family life. We found that the language used to probe about planning for pregnancy was loosely interpreted or misinterpreted, such that the term ‘planning’ has to be understood within its socio-cultural context. Any exploration of pregnancy therefore has to acknowledge the important role of infertility in reproductive health concerns. Indeed, infertility and fertility exist in a dialectical relationship of contrast, such that understanding one leads to a much greater understanding of the other [44]. Much has been written about infertility and its consequences for couples in Africa [45]–[47]; in south Asia the stigma associated with infertility can be seen as a “social response to the breakdown of a conceptual bind that conjoins the sacrament of marriage to the task of producing offspring” [48]. In Hindu families, the very notion of auspiciousness is linked to female fertility [49] across all cultural groups, however infertility is largely blamed on the woman and thought to be due to evil spirits or physiological defects in the woman [50].

The deep-rooted culturally determined beliefs of son preference in Asia are well documented. Much has been written about female foeticide, infanticide, abandonment, out-adoption, under-reporting of female births, and selective neglect of girls leading to higher death rates in South Asia in particular [51]. There are 44 million missing women in India; the sex ratio in second children if the first is a girl is even lower; strongly suggesting sex selective abortions after antenatal sex determination [52]. Given this publicly available knowledge, hearing the very real and current desire of women in Bangalore to have female children was surprising. Equally surprising was the oft repeated desire for ‘small family’ size, even a one child family. This suggests that change is occurring in socio-cultural norms and quite rapidly in certain urban settings. There is growing evidence in China that son preference is on the decline, but a recent study in both rural and urban China revealed that while son preference has weakened considerably in this generation, it has by no means disappeared [53]. Kodzi et al. [54], found the desire to stop childbearing in Ghana is influenced by reproductive life stage; events; perceptions of personal health; the household’s economic welfare; and the overall subjective cost of children. Cost of living concerns were definitely a factor in influencing the desire for small family size; however there was a strongly expressed love and appreciation of the girl child, which has not been documented before to our knowledge. The sociocultural roots of son preference even for non-resident Indians is extremely strong; in our study similarly, women were particularly pressurised to have sons by female in-laws and husbands [55]. This raises the whole question of how much if any reproductive “choice” women do have. We found that there was an ambivalence with which women regarded their own experience of reproductive choice; this began with the lack of choice or control over their marriage and extended through to their ‘planning’, family planning or pregnancy planning. The small minority of women who had had love marriages in our study had the greatest choice in planning their families, as exemplified by the young woman who became pregnant a month after her ‘love’ marriage and proudly proclaimed that she had done “planning”.

The tendency for rural South Indian women to prefer smaller babies and the relationship of this preference to dietary behaviour and quantity of food consumed during pregnancy have been well described by Nichter and Nichter [39]. We found that traditional, cultural messages about food and diet, including bewildering notions of hot and cold foods versus body habitus which have been well described, still hold meaning and relevance [56]. However curtailing diet consumed in order to have a small baby was never mentioned; the nutritional content of the food was also not a preoccupation for our mothers. A recent qualitative study of pregnant women in Mumbai similarly found that despite the knowledge that the best solution for anaemia in pregnancy was a nutritious diet, respondents did not consume appropriate diets, nor provide details of dietary regimens that would help improve iron content [57]. Breastfeeding, indeed prolonged breastfeeding, was universally practiced; other studies from this region have confirmed that prolonged breastfeeding is the norm and complementary feeding commences around four months [58]. Interestingly the ‘drink boiled water’ message, especially for baby, had translated into practice; this is clearly a change in behaviour over practices from decades ago [59]. Delayed breastfeeding, the early feeding of substitutes such as honey and *ghutti* to the newborn, which have been described in other studies from rural and urban settings [21], [60], appears to be changing in this urban setting. Cultural and religious practices from pregnancy through to the first year of life clearly have meaning, even though they were normalised (*maamuli*) and diverse in nature. Rodrigues’ study from Goa found that the failure to observe rituals and dietary practices associated with childbirth, such as the use of special diets and body massages with oil, was associated with perinatal depression [61]. Similarly women in our study who did not have a pregnancy ceremony, or were deprived of a culturally sanctioned celebration for whatever reason were unhappy.

These findings should be interpreted with certain limitations in mind. Although our study provides in-depth qualitative insight into cultural practices and beliefs throughout the perinatal period in Bangalore, it cannot be generalised beyond the urban South Indian population. Urban populations are by nature fluid and dynamic, the particular mix of cultural groups that reside in urban South India may not be similar to urban populations elsewhere. We did not specifically interview husbands, however the nature of doing field-based qualitative research in India means that the interview is with more than just the woman so that often family or friends participate, with husbands and fathers-in-law regularly attending the interviews.

In conclusion, in the great melting pot of metropolitan Bangalore, traditional and cultural practices and beliefs do strongly influence the perinatal continuum, but they are morphing and moulding according to the socio-environmental context. Kesterton and Cleland [14], from their qualitative study of birthing practices in rural Karnataka also found that movement away from traditional practices is already taking place, particularly amongst the more educated and better off. We would argue that the sometimes accusatory gaze with which ‘traditional’ practices in pregnancy and childbirth have been described, needs to be re-positioned to encompass the health and wellbeing-enhancing practices that continue to play a role for today’s mothers. There also needs to be an acknowledgement that culture is dynamic and that change is a constant. Clearly some health messages are getting through such as the need to have clean water and early attachment to the breast, while others are not. We know from research in India that community-based intervention, targeted at certain high-risk newborn-care practices, can lead to substantial behavioural modification and reduction in neonatal mortality [62]; however not all domestic behaviours change easily as our study illustrates. Further research is required to elucidate the nexus between receipt of health information and desired behaviour change. We would suggest that ethnographic research, which is multi-methodology research par excellence [30], should be given more importance both in understanding the socio-cultural contexts and in helping shape public health interventions.

We call for a greater understanding of the dynamic factors influencing and shaping the perinatal period in urban India. This includes acknowledging the critical importance of the family both in supporting and in disrupting perinatal wellbeing. It also includes acknowledging the health promoting as well as potentially harmful perinatal practices and beliefs, to result in better targeting of culturally appropriate public health interventions throughout the perinatal continuum. Behaviour change interventions to improve maternal and newborn health need to address potentially harmful practices through appropriate training of healthcare providers, changing their perceptions and attitudes, and arming them with the skills to promote positive, culturally sanctioned behaviour. Beyond merely targeting ‘practices’, healthcare providers also need to understand the socio-cultural contexts which influence beliefs and practices. This can only be achieved by involving the extended family and community in the perinatal journey.
